# Handling ‘carbon footprint’ in orthopaedics

**DOI:** 10.1308/rcsann.2023.0052

**Published:** 2024-04-02

**Authors:** S Shah, H Morris, S Thiagarajah, A Gordon, S Sharma, P Haslam, J Garcia, F Ali

**Affiliations:** ^1^ Sheffield Teaching Hospitals NHS Foundation Trust, UK; ^2^ Doncaster and Bassetlaw Teaching Hospitals NHS Foundation Trust, UK; ^3^ East Midlands North Training Rotation, UK; ^4^ Barnsley Hospital NHS Foundation Trust, UK; ^5^ Sheffield Children’s NHS Foundation Trust, UK; ^6^ Chesterfield Royal Hospital NHS Foundation Trust, UK

**Keywords:** Orthopaedics, Sustainability, Carbon footprint, Net Zero

## Abstract

**Introduction:**

The National Health Service contributes 4%–5% of England and Wales’ greenhouse gases and a quarter of all public sector waste. Between 20% and 33% of healthcare waste originates from a hospital’s operating room, and up to 90% of waste is sent for costly and unneeded hazardous waste processing. The goal of this study was to quantify the amount and type of waste produced during a selection of common trauma and elective orthopaedic operations, and to calculate the carbon footprint of processing the waste.

**Methods:**

Waste generated for both elective and trauma procedures was separated primarily into clean and contaminated, paper or plastic, and then weighed. The annual carbon footprint for each operation at each site was subsequently calculated.

**Results:**

Elective procedures can generate up to 16.5kg of plastic waste per procedure. Practices such as double-draping the patient contribute to increasing the quantity of waste. Over the procedures analysed, the mean total plastic waste at the hospital sites varied from 6 to 12kg. One hospital site undertook a pilot of switching disposable gowns for reusable ones with a subsequent reduction of 66% in the carbon footprint and a cost saving of £13,483.89.

**Conclusions:**

This study sheds new light on the environmental impact of waste produced during trauma and elective orthopaedic procedures. Mitigating the environmental impact of the operating room requires a collective drive for a culture change to sustainability and social responsibility. Each clinician can have an impact upon the carbon footprint of their operating theatre.

## Introduction

Climate change has been labelled as “the biggest global health threat of the 21st century”.^1^ In 2020, it was reported that the National Health Service (NHS) generates approximately 4%–5% of England and Wales' greenhouse gases and a quarter of all public sector waste.^2–4^ The United Nations Sustainable Development Goals strive to protect the planet and end poverty by 2030. Adequate hospital waste management contributes to success in several of the goals; in particular, health and wellbeing, clean water and sanitation, decent work and economic growth, responsible consumption and production, and climate action.^5^

In 2018, the Intergovernmental Panel on Climate Change announced that to limit global warming to 1.5°C, greenhouse gas emissions must decrease 45% by 2030 compared with 2010 and reach Net Zero by 2050.^6^ In the United Kingdom (UK), Public Health England and the NHS have estimated the health and social care climate footprint within England in 2017 to be around 6.3% of the country’s climate footprint.^7^

In 2020, the NHS launched its campaign For a Greener NHS and commissioned an expert panel to set out a practical, evidence-based and quantified path towards a “Net Zero NHS”. This report sets out a strategy and two clear targets to respond to this challenge:

• Net Zero by 2040 for the emissions the NHS controls directly
• Net Zero by 2045 for the emissions the NHS can influence.^8^

Key areas identified in this report include waste management and the procurement of lower carbon options. The main components of healthcare waste are plastic (39%–50%), textile (14%–31%), paper (11%–25%), glass (0.3%–23%), woodware (3%–20%), rubber (3%–7%), metal (0.3%–5%) and other waste (2%–19%).^9^ It has been suggested that between 20% and 33% of healthcare waste originates from a hospital’s operating room.^3^ Up to 90% of operating room waste is incorrectly sorted and sent for costly and unneeded hazardous waste processing.^10^ Contaminated or hazardous waste includes waste that has been used directly within patient care. In addition, there is often significant variation in the way in which healthcare waste is disposed of between trusts.

Although there are annual reports detailing the proportion of waste disposed of via the different waste streams, there is a lack of published data on the proportion of waste for individual operations, yet the choice of waste stream has up to a 50-fold impact on a procedure’s carbon footprint.^11,12^ The purpose of this study was to quantify the amount and type of waste produced during common trauma and elective orthopaedic arthroscopic operations across different centres.

## Methods

Prospective data collection was undertaken. All procedures were performed by senior surgeons.

Both elective and trauma operations were included (Table 1). Data were collected for ten operations of each type (e.g. total hip joint replacement, arthroscopy of knee) at each individual hospital site, provided the hospital undertook the surgery. Five hospital sites were included: one tertiary and four district general hospitals. Clinical waste created by anaesthetic colleagues was excluded.

**Table 1:** List of elective and trauma operations included in the study

Elective	Trauma
Total hip joint replacement	Dynamic hip screw
Total knee joint replacement	Cannulated screws
Total shoulder joint replacement	Hip hemiarthroplasty
Arthroscopy of knee	Intramedullary femoral nail
Arthroscopy of ankle	ORIF femur (including periprosthetic)
	ORIF tibial plateau
	Intramedullary tibial nail
	ORIF distal tibia
	External fixation/TSF tibia
	ORIF ankle
	ORIF foot
	ORIF proximal humerus
	Intramedullary humeral nail
	ORIF distal humerus/proximal humerus
	TENS nail forearm
	ORIF forearm
	ORIF distal radius
	MUA ± K-wire distal radius
	ORIF hand
	MUA ± K-wire hand

MUA = Manipulation under Anaesthetic; ORIF = Open Reduction and Internal Fixation; TENS = titanium elastic nail; TSF = Taylor Spatial Frame

The intraoperative waste from the surgeries was measured. The waste was separated into four bags: clean paper, contaminated paper, clean plastic and contaminated plastic. Contaminated waste is any that has been used directly in patient care; it does not include product wrappers but would include each item in a multipack surgical set, even if that item had not been used. Paper and plastic were measured because they comprise the largest amount of waste; disposable gowns and drapes are manufactured from plastic. At the end of each procedure, each bag of waste was weighed using a theatre scale DIGI® DS-502 (Marsden Weighing Machine Group Ltd, Rotherham, UK). The mean measurement of waste was taken for each procedure from each centre.

Subsequently, estimated annual carbon emissions of the waste disposal for each procedure were calculated. This calculation used retrospective case numbers from pre-COVID surgery for the year 2019–2020, identified by searching with clinical codes, and data provided by each waste contractor on the carbon emissions, cost and way in which they process the waste.

Following this, hospital site A undertook a trial in which 1,000 reusable surgical gowns were rented for four weeks and used in theatre in place of disposable gowns. The gowns were laundered by a commercial company that already held a contract with the NHS. The carbon footprint for 1,000 uses of reusable and disposable surgical gowns was calculated using data based on the Overcash study of life-cycle assessments for surgical gowns.^13^ The costs for using reusable and disposable gowns over the 2019–2020 period were calculated based on data supplied by the hospital.

## Results

### Trauma and elective waste

Table 1 lists the operations included in the study, which were divided into elective or trauma. Waste was measured over ten procedures for each operation. The range of total paper and plastic waste within the two groups, and for selected elective and trauma operations within the study, is demonstrated in Table 2. The mean waste for four trauma and four elective procedures is also shown.

**Table 2:** Range of paper and plastic waste for elective and trauma surgery

Procedure type	Paper waste (kg)		Plastic waste (kg)	
	Mean	Range	Mean	Range
Trauma		0.4–2.2		2.4–12.6
Dynamic hip screw	0.9	0.4–0.9	11.4	6.9–12.6
Hip hemiarthroplasty	1.2	0.8–1.4	10.8	8.9–12.2
ORIF ankle	1.0	0.8–1.2	7.4	5.9–9.7
ORIF distal radius	0.8	0.6–0.8	6.3	4.9–7.1
Elective		1.1–3.2		3.3–16.5
Hip arthroplasty	2.4	1.8–2.9	11.8	6.6–16.5
Knee arthroplasty	2.4	1.8–2.8	10.7	6.5–13.8
Knee arthroscopy	1.3	1.1–1.6	8.6	3.4–9.6
Shoulder arthroscopy	1.4	1.2–2.3	10.6	3.3–15.5

ORIF = open reduction and internal fixation

Table 3 shows four elective and four trauma operations commonly undertaken at each hospital site and lists the mean total plastic waste over ten of these procedures at each hospital site.

**Table 3:** Mean total plastic waste (kg) for selected operations across hospital sites

Procedure	Hospital site					
	A	B	C	D	E	
Trauma						
Dynamic hip screw	11.2	9.6	12.1	11.6	12.2	
Hip hemiarthroplasty	10.6	9.2	11.4	10.9	11.8	
ORIF ankle	6.9	6.2	7.3	8.6	7.8	
ORIF distal radius	6.1	6.2	6.4	6.2	6.6	
Elective						
Hip arthroplasty	11.2	10.2	11.9	11.8	13.9	
Knee arthroplasty	9.9	9.8	11.2	10.8	12.2	
Knee arthroscopy	9.1	5.8	8.8	5.8	10.4	
Shoulder arthroscopy	10.1	7.2	9.2	7.6	15.1	

ORIF = open reduction and internal fixation

### Annual plastic waste, costs for waste disposal and carbon emissions

Mean total plastic waste per procedure in orthopaedics was calculated for each hospital.

The number of procedures undertaken at each site in 2019–2020 was obtained and used to calculate the annual waste for these procedures. The cost for each hospital site to process the plastic waste was calculated. This is shown in Table 4. Of note, each hospital site uses a separately negotiated waste contract and contractor. The processing cost per tonne of plastic waste at each site is also shown.

**Table 4:** Plastic waste, disposal costs and carbon emissions across hospital sites

	Hospital site				
	A	B	C	D	E
Total number of procedures	2,826	2,921	4,981	2,791	12,562
Plastic waste per procedure (kg)	9	6	9	10	12
Total annual plastic waste (tonnes)	25.4	17.5	44.8	27.9	150.7
Processing cost/tonne (£)	580	450	750	746	520
Total cost incurred (£)	14,752	7,887	33,622	20,821	78,387
Total annual carbon emissions (tonnes)	6.1	3.6	10.8	6.7	49.0

The hospitals in the study separated clean plastic and paper waste put into the same bag. Unfortunately, the hospital waste contractors provide a recycling service but are unable to separate combined waste into its individual plastic and paper components and so this waste is incinerated with energy recapture. Contaminated waste was disposed of either to landfill or by incineration. The carbon emissions of processing a tonne of waste via these methods was taken from information provided by each waste contractor. Carbon emissions were calculated using these figures for the disposal of plastic, and the data are given in Table 4.

Hospital site B used reusable gowns and disposable drapes for most of their day case procedures and disposable gowns and drapes for non-day case procedures. The remaining hospital sites used disposable gowns and drapes for all procedures. Of note, hospital site E used a double-draping system for elective procedures. This involves utilising disposable drapes in the anaesthetic room and then re-draping the patient when they are in the main theatre.

### Reusable vs disposable gowns

Finally, published work by Overcash on life-cycle assessments of surgical gowns was used to estimate the difference in carbon emissions of using 1,000 reusable gowns, for 60 uses per gown, and 1,000 disposable gowns over a four-week period at hospital site A. The figures taken from the work by Overcash are shown in Table 5. Of note, International Organization for Standardization standards allow for 75 uses per gown, although the average use rate is lower because of losses, damage and so on. The Overcash work had a gown use rate of 60 cycles to account for this.^13^ The use of reusable gowns potentially results in a 66% reduction in carbon emissions.

**Table 5:** Carbon footprint of 1,000 gowns

	Carbon footprint (kgCO_2_)	
	Reusable (>60 uses)	Disposable
Gown manufacture and supply chain	143.0	1,495.0
Packaging and supply chain	76.7	121.0
Laundry	278.0	0
Sterilisation	19.8	6.3
Use phase transport	38.7	3.5
End of life	1.4	10.9
Total carbon footprint	557.6	1,636.7
Carbon footprint per gown	0.56	1.64

Costings were requested from the trust of hospital site A and used to estimate cost savings over a year using the number of operative procedures within the trust for the period 2019–2020. During 2019–2020, some 62,730 gowns were used at a total cost of £60,698.67. In 2019–2020, the cost per use for a disposable gown was £0.97 and the cost per use for a reusable gown was £0.75. If all 62,730 gowns that were used were reusable, the annual cost would be £47,214.78. This represents a cost saving of £13,483.89. The trust also estimated costs based on uplift predictions for 2022–2023. The cost for using disposable gowns would be £78,887.03 and the cost for using reusable gowns is £72,757.76. This represents a potential cost saving of £6,129.27.^14^

## Discussion

This study aligns with the Royal College of Surgeons England (RCS England) Sustainability in Surgery Strategy 2021 and the plastics reduction pledge of the NHS “Net Zero” report, highlighting the importance of addressing waste and waste processing in producing a greener operating theatre.^8,15^ It has been estimated that over half of the global population are at risk from environmental, occupational or public health threats resulting from incorrectly treated healthcare waste.^16^

There are several ways of reducing clinical waste and particular attention has been paid to implementing a circular healthcare economy model: reducing, reusing, recycling.

• Reducing what is consumed can be achieved by identifying whether the item needs to be opened for the set. Do we need to use multipack options for surgical tools when single-packed items may suffice? Reducing the amount of plastic used is critical because the recycling and processing of clean plastic waste generates a greater carbon footprint than the processing of clean paper waste, even though the financial cost was the same to the trust in the contracts we analysed.^17^
• Reusing items, and selecting items that can be laundered, reduces the production and subsequent consumption of items; for example, the use of reusable kidney bowls, light handles and drapes.
• Recycling allows for a reduction in waste going forward to incineration and landfill.
• If waste must be disposed of, utilise methods that allow for energy harvested and reuse over landfill.

As clinicians, we are at the end of the medical device life cycle. Medical device designers, manufacturers and procurement also have a role to play in waste production. Over 80% of the environmental impact of a product is determined at the design stage.^18^ Using resources efficiently by considering the use of renewable and sustainable raw materials, reducing the amount of resources consumed in a device’s manufacture, delivery and operation, and waste minimisation all affect the environmental impact. Medical devices should be designed for circularity (a product suitable for several life cycles), durability, disassembly and reuse. Finally, product life should be extended to keep the product in use for the greatest number of safe-use cycles rather than disposing of it after a single use.

Reducing our consumption is critical. There has already been interest in this area with consideration given to what is in a multipack in theatre. An American study including wide-awake hand surgery cases had clinicians involved in deciding what was in the multipack with a subsequent 13% reduction in waste per case and a cost saving of $125.^19^ A further unit removed 15 items from their disposable plastic pack and seven from the hand pack, with an estimated annual saving of US$17,381.05 in the unit from these changes alone.^20^

Plastic constituted the greatest amount of waste, in line with other studies and our expectations. Despite the hospitals separating out clean plastic waste, it was combined with clean paper waste and none of the waste contractors provide a recycling service that can separate the two and recycle, and so this waste is incinerated with energy recapture. It is likely that other trusts have a similar issue where the perception of recycling in theatres is not realised because of a lack of sorting in the hospital or a lack of facilities at the waste contractors.

In our study, primary hip arthroplasties generated a mean of 11.8kg of plastic waste per procedure. This is comparable with previous published literature; a two-hospital study in the East of England in 2011 found 12.1kg of waste was generated for a total hip arthroplasty, of which 6% was contaminated and not appropriate for recycling.^21^ More recently, in 2022, a study measuring total hip arthroplasty waste in Newcastle upon Tyne found that the average amount of waste produced for a single total hip arthroplasty was 10.9kg.^22^ Both studies reported that there was the potential to recycle a greater percentage of the waste, without incurring additional harm from hazardous waste, compared with the current practice, and suggested that staff education is critical to ensure waste is being disposed of accurately.

We noted a large range in the plastic waste measured for different procedures; for example, 9.9kg for hip arthroplasty, 7.3kg for knee arthroplasty and 12.2kg for shoulder arthroscopy. Trauma surgery also generated differences, although these were smaller: 5.7kg for a dynamic hip screw, 3.3kg for a hip hemiarthroplasty and 3.8kg for an ankle fixation. One reason identified was the surgeon’s preference for draping patients. There was significant discrepancy between different surgeons undertaking the same operation. The second factor identified was whether reusable or disposable gowns were used.

The results show that hospital site B had the lowest mean total plastic waste. This site uses reusable gowns and disposable drapes for most of their day case procedures, such as knee or shoulder arthroscopy and distal radius fixation, and disposable gowns and drapes for non-day case procedures such as hip and knee arthroplasty, and hip hemiarthroplasty. Taking this into account, their figures were still generally lower even for those surgeries where disposable textiles were used. This may be because of an awareness and culture within the hospital towards sustainability.

Interestingly, one surgeon at hospital site B uses reusable gowns and drapes because of an allergy to the disposable gowns. Previous unpublished work at this hospital site has demonstrated that the amount of textile waste produced in arthroscopic surgery is 6kg.

Hospital site E uses a double-draping system for elective procedures. This involves utilising disposable drapes in the anaesthetic room and then re-draping the patient when they are in the main theatre. The mean total plastic waste generated for these procedures at this hospital site was the greatest across all the sites included in the study, reinforcing the importance of surgeon preference for draping and its impact on waste generated.

The use of reusable or disposable drapes and gowns appeared to be a key factor in the differences in waste produced between hospital sites. Work is currently underway via the: Sustainability in Surgery Strategy and partners of The Centre for Sustainable Healthcare's Green Surgery Challenge Oversight Committee to identify the ways in which surgery can be made greener and more environmentally friendly. One of the areas being addressed within the surgical community is waste production, and this includes whether medical textile waste, such as gowns and drapes, can be minimised without incurring patient harm. There have been no randomised controlled trials to assess the incidence surgical site infections (SSIs) with reusable or disposable gowns and drapes. Despite great enthusiasm being shown for investigating the best types of fabric for a surgical gown, replication of results in vivo and in vitro often differ.^23^ A recent review of SSIs in orthopaedic and spine surgery found no available evidence to support a difference between reusable and disposable gowns and drapes, and called for further research.^24^ World Health Organization Global Guidelines for the Prevention of Surgical Site Infection recommend the use of reusable or disposable gowns and drapes, and acknowledge the paucity of evidence in this domain.^25^ Given the lack of evidence directly comparing the incidence of SSIs with reusable and disposable gowns, we cannot conclusively state that the two are equivalent at preventing SSIs in clean surgery; it is suspected, however, that there is little difference in clean-contaminated surgery and no difference in dirty or contaminated surgeries.

Our pilot study substituting disposable gowns for reusable ones demonstrated a 66% reduction in carbon emissions when calculated using data published by Overcash.^13^ This was by no means an in-depth life-cycle assessment. However, most life cycle studies focusing on medical devices in surgical settings compare single-use items with their equivalent reusable solutions, and find that reusable options generally perform better across a broad range of environmental impact categories, including climate change.^26^ For example, a recent life-cycle analysis of scrub suits found that a reusable scrub suit has a 31% reduced impact on climate change compared with a disposable scrub suit system. This study considered a day’s use of 1.8 scrub suits and found these had a 62% lower impact when reusables were used compared with disposables.^27^

Recently, a team based in London replaced 3,051 disposable gowns with reusable ones over a six-month period and demonstrated a saving of 3.292 tonnes of CO_2_ emissions and a cost saving of £366 from reductions in waste disposal and standard gown use.^28^ Scaling these figures up to 62,730 gowns, the cost savings in this study would be £7,525.13. Our cost saving of £13,483.89 was based on 2019–2020 data. Costs have increased because of the pandemic and using uplifted costs, our savings reduce to £6,129.27, which is similar to the London-based team’s findings when taking into account that hospitals negotiate their own waste disposal contracts.

### Study limitations

This study did not assess the waste created by anaesthetic procedures nor did it assess the total amount of waste per procedure, the percentage that was recycled or the percentage sent to landfill. Surgeons were aware that the study was being undertaken and as such the results may show bias. Although it involves a small sample size of five hospitals, raw data in the published field are still relatively scarce and this could be a benchmark for others to use and add to.^29^ Figures for carbon emission reduction were based upon the Overcash study, which makes several assumptions on gown use and is based on US rather than UK data.^13^

Further research should be directed at assessing waste production and processing. Some plastics are recycled more readily than others, and further investigation into the type of plastic disposed of during procedures would be valuable to assess whether we are using appropriate plastics in our packaging types to create a greener operating environment. Accurate and in-depth life-cycle assessments are costly and time-consuming but we would benefit from producing life-cycle assessments of medical devices, such as surgical gowns, using items in circulation and current practices for laundering and disposal. Finally, attention should be paid to qualitative work exploring the education and beliefs of staff concerning the recycling of theatre waste; educating staff on what to recycle has demonstrated an increase in the overall recycling of theatre waste.^30^ Exploring surgeons’ beliefs regarding the draping of patients for procedures and comparing this with infection rates would also be valuable for providing an understanding behind some of the discrepancies in practice we encountered.

## Conclusions

This study sheds new light on the environmental impact of waste produced in trauma and elective orthopaedic procedures. Given the national and international targets, it is timely to draw this to the attention of the clinician. Mitigating the environmental impact of the operating room requires a collective drive for a culture change to sustainability and social responsibility. At a national level, consideration should be given to the design, manufacture and procurement of appropriate products with attention paid to environmentally sustainable options. Clinicians should be working with hospital management and procurement to create an environmentally more sustainable operating theatre, and consideration should be given to the methods in which waste is managed.
